# Cell kinetics: an independent prognostic variable in stage II melanoma of the skin.

**DOI:** 10.1038/bjc.1990.386

**Published:** 1990-11

**Authors:** A. Costa, R. Silvestrini, G. Mezzanotte, M. Vaglini, E. Grignolio, C. Clemente, N. Cascinelli

**Affiliations:** Oncologia Sperimentale C, Instituto Nazionale Tumori, Milan, Italy.

## Abstract

The prognostic role of cell kinetics (expressed as 3H-thymidine labelling index, 3H-TdR LI) was assessed on 145 patients with pathologic stage II melanoma subjected only to therapeutic lymph node dissection. The 3H-TdR LI determined on metastatic nodes was related to relapse-free survival and to survival. In particular, 3-year relapse-free survival was significantly different from patients with slowly and rapidly proliferating melanomas (40% vs 22%, P = 0.007), and this finding was consistently found for overall survival (68% vs 46%, P = 0.007). Moreover, in patients with high 3H-TdR LI tumours, the risk of relapse and death within the first year from lymphadenectomy was two-fold that of patients with low 3H-TdR LI tumours. Multiple regression analysis showed that 3H-TdR LI retained its prognostic significance on relapse-free and on overall survival even when the number of involved nodes and type of nodal metastases were considered. Present findings suggest that 3H-TdR LI can contribute to select high-risk stage II melanoma patients.


Br. J. Cancer (1990), 62, 826-829 © Macmillan Press Ltd., 1990

### Cell kinetics: an independent prognostic variable in stage II melanoma of the skin

A. Costa, R. Silvestrini, G. Mezzanotte, M. Vaglini, E. Grignolio, C. Clemente & N. Cascinelli

*Oncologia Sperimentale C, Istituto Nazionale Tumori, Via Venezian 1, 20133 Milan, Italy.*

**Summary** The prognostic role of cell kinetics (expressed as $^{3}$H-thymidine labelling index, $^{3}$H-TdR LI) was assessed on 145 patients with pathologic stage II melanoma subjected only to therapeutic lymph node dissection. The $^{3}$H-TdR LI determined on metastatic nodes was related to relapse-free survival and to survival. In particular, 3-year relapse-free survival was significantly different from patients with slowly and rapidly proliferating melanomas (40% *vs* 22%, $P=0.007$), and this finding was consistently found for overall survival (68% *vs* 46%, $P=0.007$). Moreover, in patients with high $^{3}$H-TdR LI tumours, the risk of relapse and death within the first year from lymphadenectomy was two-fold that of patients with low $^{3}$H-TdR LI tumours. Multiple regression analysis showed that $^{3}$H-TdR LI retained its prognostic significance on relapse-free and on overall survival even when the number of involved nodes and type of nodal metastases were considered. Present findings suggest that $^{3}$H-TdR LI can contribute to select high-risk stage II melanoma patients.

Prognosis in patients with stage II melanoma is related to features of the primary tumour and of nodal metastases (Balch *et al.*, 1981; Cascinelli *et al.*, 1984; Roses *et al.*, 1985; Koh *et al.*, 1986; Kissin *et al.*, 1987). Survival is inversely related to the number of positive nodes and also decreases when perilymph node invasion is present (Balch *et al.*, 1981; Rayner *et al.*, 1981; Callery *et al.*, 1982; Cascinelli *et al.*, 1984). However, it has also been emphasised that nodal metastases are indicators, not determinants of survival in pathologic stage II melanoma patients (Cady, 1984).

Cell kinetics, studied by different approaches, has emerged as an important indicator of biologic aggressiveness (Bauer *et al.*, 1987; Kallioniemi *et al.*, 1987; Quirke *et al.*, 1987; Griffin *et al.*, 1988; Hall *et al.*, 1988; Bouzubar *et al.*, 1989). In particular, the $^{3}$H-thymidine labelling index ($^{3}$H-TdR LI) has been shown to be a prognostic factor whose role remains also in the presence of other clinico-pathologic prognostic factors (Meyer *et al.*, 1983; Silvestrini *et al.*, 1986, 1989; Chauvel *et al.*, 1988).

Little attention has been given to cell kinetics in malignant melanoma (Shirakawa *et al.*, 1970; Hagemann & Schiffer, 1971; Newburger *et al.*, 1980; Hansson *et al.*, 1982; Costa *et al.*, 1987). The data available on preliminary series of stage II melanoma patients strongly indicate the potential clinical usefulness of cell kinetics as a prognostic marker (Hansson *et al.*, 1982; Costa *et al.*, 1987). However, the relative contribution of cell kinetics in relation to information given by other morphologic and pathologic features has been never analysed.

In this follow-up study, we report on a group of 166 patients with pathologic stage II melanoma of the skin to substantiate, in a larger series with a longer follow-up, our preliminary findings. However, we investigated the possible association of cell kinetics with established clinico-pathologic parameters and we estimated the different contribution of all the variables on the clinical course, in terms of relapse-free interval and overall survival, by means of a multiple regression analysis.

The main basic limitation in starting the study was the impossibility to perform the cell kinetics determination on the primary tumour, which is completely reserved for the pathologist for Clark's (1969) and Breslow's (1970) histologic microstaging. This constraint was overcome by using the metastatic nodal lesion. This decision is quite compatible with the microscopic examination of autoradiograms, which allows us accurately to score and consider only tumour cells.

Correspondence: R. Silvestrini.
Received 14 March 1990; and in revised form 22 May 1990.

### Material and methods

### Case series

The study was carried out on 166 patients with stage II melanoma of the skin. Stage was assessed by conventional clinical, radiological and radioisotopic examinations. All patients underwent therapeutic radical lymph node dissection at the Cancer Institute of Milan from November 1981 to September 1987. The primary tumour had been previously removed in 121 cases, and in 45 cases it was removed simultaneously with nodal dissection. No other treatment was given until new disease manifestation was documented. Sixty-six patients were females and 100 males. The median age at the time of node dissection was 47 years (range 18-79 years).

A histopathological examination was performed to evaluate the number and type of nodal metastases. Sixty-six patients had one positive node (N + ), 41 patients had two, 12 patients had three and 47 patients had more than three positive nodes. The group of patients defined by the type of nodal involvement was numerically unbalanced. In fact, only 13 patients showed partial nodal invasion, 48 patients massive invasion still confined within the capsule, and 105 patients invasion beyond the capsule. This was due to the impossibility to recognise by gross examination slightly or partially involved metastases at the time of node dissection.

### Cell kinetic determination

Cell kinetics was defined as *in vitro* $^{3}$H-TdR LI on fresh pathologic lymph nodes immediately after dissection, as previously described (Costa *et al.*, 1987). Briefly, small fragments were incubated with $^{3}$H-thymidine (6 $\mu$Ci ml$^{-1}$, specific activity, 5 Ci mmol$^{-1}$; Radiochemical Centre, Amersham, UK) in culture medium for 1 h at 37°C. Autoradiography was performed on histologic sections according to the stripping film technique (Kodak AR10, Kodak, London, UK), and the samples were stained with haematoxylin and eosin. The $^{3}$H-TdR LI was determined by scoring at least 3,000 tumour cells per sample. The determination of $^{3}$H-TdR LI on the sections from different samples of the same tumour showed a median coefficient of variation of 25%.

### Statistical methods

Comparison of the $^{3}$H-TdR LI values among the different subsets identified by the various prognostic factors was done by the Wilcoxon rank sum test. The Bonferroni procedure was adopted to allow for multiple comparisons. The two major end-points, relapse-free survival (RFS) and overall survival, were computed from the time of node dissection until clinically evaluated relapse or death on 145 patients with an adequate follow-up. The observation times ranged from 1 to 75 months, and the median follow-up was 25 months. First of all, a univariate analysis was carried out to estimate RFS and survival curves relative to the different modalities of each prognostic variable by means of the Kaplan–Meier product-limit method (Kaplan & Meier, 1958). Comparisons among curves were accomplished by resorting to the log rank test (Mantel, 1966). The hazard function was estimated according to the approach proposed by Simes and Zelen (1985). Six-month intervals were chosen to calculate the hazard estimates.

The joint effect of the variables, possibly influencing prognosis, was investigated by a Cox's multiple regression model (Cox, 1972).

The role of $^3$H-TdR LI was investigated after classifying tumours in two classes with different proliferative rates. the value of 8%, which represents the mean value of the distribution of this variable, was used to discriminate slowly and rapidly proliferating melanomas. Other cut-off levels were tested by means of the Kaplan–Meier product-limit method, and the log rank test was used to assess differences in RFS and survival between subgroups. $\chi^2$ values with one degree of freedom were calculated with the corresponding $P$ values, and the highest $\chi^2$ value was observed at a $^3$H-TdR LI cut-off of 8%.

### Results

$^3$H-TdR LI values defined for the overall series of 166 nodal lesions were highly skewed, with a mean value of 8.6% and a range from 0.3 to 31%, in agreement with our previous results (Costa *et al.*, 1987). Cell kinetics was analysed in relation to the clinico-pathological features considered prognostic factors in stage II melanoma patients (Table I). $^3$H-TdR LI was not related to age or sex of the patient, to number of positive nodes, or to type of metastases.

The overall 3-year RFS and survival for the series of 145 patients, for whom cell kinetic and follow-up information was available, were 32% and 59%, respectively. This finding is in agreement with other clinical reports on larger series of stage II melanoma patients (Balch *et al.*, 1981; Cascinelli *et al.*, 1984; Kissin *et al.*, 1987). The analysis as a function of $^3$H-TdR LI showed significant differences in the probability of RFS between patients with slowly and rapidly proliferating tumours (40% *vs* 22%, $P = 0.007$) (Figure 1a). The survival curves were also significantly different (68% *vs* 46%, $P = 0.007$) (Figure 1b). Relapse and death hazard over time showed similar patterns for the two kinetics subsets of patients (Figure 2), with a peak of risk from 6 to 12 months from lymphadenectomy. The hazard was higher for patients with rapidly proliferating tumours than for those with slowly proliferating tumours for the whole follow-up period.

**Table I** Relationship between cell kinetics and clinico-pathologic features in stage II melanoma

| | *No. of cases* | *Labelling index (%)* *Mean* | *Range* |
|---|---|---|---|
| *Patient age (years)* | | | |
| ≤40 | 55 | 8.5 | 0.9–31.1 |
| <40 | 111 | 8.6 | 0.3–29.6 |
| *Sex* | | | |
| Males | 100 | 8.3 | 0.3–29.6 |
| Females | 66 | 9.0 | 0.8–31.1 |
| *Nodal status* | | | |
| N+ ≤2 | 107 | 9.0 | 0.6–29.6 |
| N+ >2 | 59 | 7.8 | 0.3–31.1 |
| *Type of nodal metatasis* | | | |
| Intracapsular | 61 | 7.6 | 0.8–24.8 |
| Extracapsular | 105 | 9.0 | 0.3–31.1 |

Univariate analysis of 3-year RFS and survival as a function of number of positive nodes and type of nodal invasion was performed on the two subsets with less or more than two positive nodes owing to the unbalanced number of cases within the different categories. Patients with partial or massive nodal involvement were considered together. When

**Figure 1** Clinical outcome as a function of $^3$H-TdR LI of nodal metastases in a series of 145 patients with stage II melanoma of the skin. ---, low $^3$H-TdR LI; —, high $^3$H-TdR LI. **a**, relapse-free survival; **b**, survival.

**Figure 2** Estimated hazard function of relapse and death (per 100 persons per 6 months) for patients with slowly (---) and rapidly (—) proliferating tumours.

singly tested, number of positive nodes was associated to clinical outcome. Patients with $\leqslant 2$ N + showed a higher probability of RFS (43% vs 13%, $P = 0.0004$) and survival (66% vs 46%, $P = 0.04$) than patients with $> 2$ N + (Figure 3a,b). Moreover, the probability of RFS at 3 years was significantly lower for patients with extracapsular diffusion of disease (27%) than for patients with intracapsular disease (44%; $P = 0.03$) (Figure 4a). Within the latter group, a different RFS was observed for patients with partial or massive nodal involvement. No statistically significant difference was observed among the corresponding survival curves. (Figure 4b). Age and sex of patients did not affect RFS or survival.

Multiple regression analysis was carried out to evaluate the joint effect of prognostic factors on RFS and survival. Age and sex were excluded from the evaluation because, by single factor analysis, they were clearly not associated with RFS and survival. The $^3$H-TdR LI was added to the regression models including the two pathologic variables, i.e. number of positive nodes and type of nodal invasion (Table II). With regard to RFS, the hazard ratio was maximum for number of positive nodes. $^3$H-TdR LI, as in the univariate analysis, retained its statistical significance and provided a prognostic contribution to the other variables considered in the model (likelihood ratio test: $\chi^2 = 5.74$, 1 d.f., P = 0.017). Type of nodal invasion did not reach the significance level. $^3$H-TdR LI became the most important prognostic indicator of survival compared to the pathologic variables (likelihood ratio test: $\chi^2 = 7.23$, 1 d.f., $P = 0.007$).

**Figure 3** Clinical outcome as a function of number of positive nodes in a series of 145 patients with stage II melanoma of the skin. ---, $\leqslant 2$ N+; —, $> 2$ N+. **a**, relapse-free survival; **b**, survival.

**Figure 4** Clinical outcome as a function of type of nodal invasion in a series of 145 patients with stage II melanoma of the skin. ---, intracapsular invasion; —, extracapsular invasion. **a**, relapse-free survival; **b**, survival.

### Discussion

Tumour thickness is the most important factor in predicting the risk of nodal metastases in stage I melanoma patients, but it no longer has any prognostic value once nodal metastases have developed. At such a time, in stage II patients, nodal status becomes the most important determinant of evolution, i.e. the second step of melanoma life is mainly conditioned by number of involved nodes and type of nodal metastases (Balch *et al.*, 1981; Rayner *et al.*, 1981; Callery *et al.*, 1982; Cascinelli *et al.*, 1984). This finding has been confirmed in the present series of patients: 3-year RFS and survival were significantly lower for patients with more than two positive nodes and massive invasion, regardless of diffusion beyond the capsule.

As observed for other human tumours (Meyer *et al.*, 1983; Tubiana *et al.*, 1984; Silvestrini *et al.*, 1986, 1989*a,b*; Chauvel *et al.*, 1988), cell kinetics varies widely from patient to patient, and there is no evident relationship between $^3$H-TdR LI of metastatic lesions and clinico-pathologic features considered to be prognostic factors in stage II melanoma patients.

The present study confirms the previous finding on the value of cell kinetics as a prognostic factor in stage II melanoma patients subjected to nodal dissection (Hansson *et al.*, 1982;

**Table II** Final model of Cox's regression analysis relative to relapse-free and overall survival

| | *RFS* | | *Survival* | |
|---|---|---|---|---|
| *Variable* | *Hazard ratio* | *95% confidence limits* | *Hazard ratio* | *95% confidence limits* |
| *No. of positive nodes* | | | | |
| $>2$ vs $\leqslant 2$ | 2.0 | 1.3–3.1 | 1.9 | 1.0–3.6 |
| *Type of nodal metastasis* | | | | |
| Extra vs intracapsular | 1.3 | 0.8–2.1 | 1.1 | 0.6–2.2 |
| *$^3$H-TdR LI* | | | | |
| High vs low | 1.7 | 1.1–2.6 | 2.3 | 1.3–4.1 |

Costa *et al.*, 1987), even after making allowance for all the other known prognostic factors. In fact, multiple regression analysis showed that $^{3}$H-TdR LI retained its importance as an indicator of RFS and became the most important indicator of survival even in the presence of number of involved nodes and type of nodal metastases, at least for the first 3 years after surgery. Our findings, which reproduce those reported for other tumour types (Meyer *et al.*, 1983; Tubiana *et al.*, 1984; Silvestrini *et al.*, 1986, 1989*a*,*b*; Chauvel *et al.*, 1988), indicate that, even for stage II melanoma patients, cell kinetics should improve the assessment of prognosis.

### References

BALCH, C.M., SOONG, S.J., MURAD, T.M., INGALLS, A.L. & MADDOX, W.A. (1981). A multifactorial analysis of melanoma. III. Prognostic factors in melanoma patients with lymph node metastases (stage II). *Ann. Surg.*, **81**, 377.

BAUER, K.D., LINCOLN, S.T., VERA-ROMAN, J.M. & 5 others (1987). Prognostic implications of proliferative activity and DNA aneuploidy in colonic adenocarcinomas. *Lab. Invest.*, **57**, 329.

BOUZUBAR, N., WALKER, K.J., GRIFFITHS, K. & 5 others (1989). Ki67 immunostaining in primary breast cancer: pathological and clinical associations. *Br. J. Cancer*, **59**, 943.

BRESLOW, A. (1970). Thickness, cross-sectional areas and depth of invasion in the prognosis of cutaneous melanoma. *Ann. Surg.*, **172**, 902.

CADY, B. (1984). Lymph node metastases. Indicators, but not governors of survival. *Arch. Surg.*, **119**, 1067.

CALLERY, C., COCHRAN, A.J., ROE, D.J. & 5 others (1982). Factors prognostic for survival in patients with malignant melanoma spread to the regional lymph nodes. *Ann. Surg.*, **196**, 69.

CASCINELLI, N., VAGLINI, M., NAVA, M. & 6 others (1984). Prognosis of skin melanoma with regional node metastases (stage II). *J. Surg. Oncol.*, **25**, 240.

CHAUVEL, P., COURDI, A., GIOANNI, J., VALLICIONI, J., SANTINI, J. & DEMARD, F. (1988). The labeling index: a prognostic factor in head and neck carcinoma. *Radiother. Oncol.*, **547**, 1.

CLARK, W.H., FROM BERNARDINO, E.A. & MIHM, M.C. (1969). The histogenesis and biologic behavior of primary human malignant melanomas of the skin. *Cancer Res.*, **29**, 705.

COSTA, A., SILVESTRINI, R., GRIGNOLIO, E., CLEMENTE, C., ATTILI, A. & TESTORI, A. (1987). Cell kinetics as a prognostic tool in patients with metastatic malignant melanoma of the skin. *Cancer*, **60**, 2797.

COX, D.R. (1972). Regression models and life tables. *J. R. Stat. Soc.*, **34**, 187.

GRIFFIN, N.R., HOWARD, M.R., QUIRKE, P., BRIEN, C.J.O., CHILD, J.A. & BIRD, C.C. (1988). Prognostic indicators in centroblastic-centrocytic lymphoma. *J. Clin. Pathol.*, **41**, 866.

HAGEMANN, R.F. & SCHIFFER, L.M. (1971). Cell kinetic analysis of a human melanoma in vitro and in vivo-vitro. *J. Natl Cancer Inst.*, **47**, 519.

HALL, P.A., RICHARDS, M.A., GREGORY, W.M., D'ARDENNE, A.J., LISTER, T.A. & STANSFELD, A.G. (1988). The prognostic value of Ki67 immunostaining in non-Hodgkin's lymphoma. *J. Pathol.*, **154**, 223.

HANSSON, J., TRIBUKAIT, B., LEWENSOHN, R. & RINGBORG, U. (1982). Flow cytofluorometric DNA analyses of metastases of human malignant melanomas. *Anal. Quant. Cytol.*, **4**, 99.

KALLIONIEMI, O.P., HIETANEN, T., MATTILA, J., LEHTINEN, M., LAUSLAHTI, K. & KOIVULA T. (1987). Anueploid DNA content and high S-phase fraction of tumour cells are related to poor prognosis in patients with primary breast cancer. *Eur. J. Cancer Clin. Oncol.*, **23**, 277.

KAPLAN, E.L. & MEIER, P. (1958). Nonparametric estimation from incomplete observations. *J. Am. Stat. Assoc.*, **53**, 457.

KISSIN, M.W., SIMPSON, D.A., EASTON, D., WHITE, H. & WESTBURY, G. (1987). Prognostic factors related to survival and groin recurrence following therapeutic lymph node dissection for lower limb malignant melanoma. *Br. J. Surg.*, **74**, 1023.

KOH, H.K., SOBER, A.J., DAY, C.L. & 8 others (1986). Prognosis of clinical stage I melanoma patients with positive elective regional node dissection. *J. Clin. Oncol.*, **4**, 1238.

MANTEL, N. (1966). Evaluation over survival data and two new rank order statistics araising in its consideration. *Cancer Chemother. Rep.*, **50**, 163.

MEYER, J.S., FRIEDMAN, E., MCCRATE, M.M. & BAUER, W.C. (1983). Prediction of early course of breast carcinoma by thymidine labeling. *Cancer*, **51**, 1879.

NEWBURGER, A.E. & WEINSTEIN, G. (1980). Cell proliferation patterns in human malignant melanoma, in vivo. *Cancer*, **46**, 313.

QUIRKE, P., DIXON, M.F., CLAYEN, A.D. & 4 others (1987). Prognostic significance of DNA aneuploidy and cell proliferation in rectal adenocarcinomas. *J. Pathol.*, **151**, 285.

RAYNER, C.R. (1981). The results of node resection for clinically enlarged lymph nodes in malignant melanoma. *Br. J. Plast. Surg.*, **34**, 152.

ROSES, D.F., PROVET, J.A., HARRIS, M.N., GUMPORT, S.L. & DUBLIN, N. (1985). Prognosis of patients with pathologic stage II cutaneous malignant melanoma. *Ann. Surg.*, **201**, 103.

SHIRAKAWA, S., LUCE, J.K., TANNOCK, I. & FREI, E. III (1970). Cell proliferation in human melanoma. *J. Clin. Invest.*, **49**, 1188.

SILVESTRINI, R., DAIDONE, M.G., DI FRONZO, G., MORABITO, A., VALAGUSSA, P. & BONADONNA, G. (1986). Prognostic implication of labeling index versus estrogen receptors and tumor size in node-negative breast cancer. *Breast Cancer Res. Treat.*, **7**, 161.

SILVESTRINI, R., COSTA, A., GIARDINI, R. & 4 others (1989*a*). Prognostic implications of cell kinetics, histopathology and pathologic stage in non-Hodgkin's lymphomas. *Hematol. Oncol.*, **7**, 411.

SILVESTRINI, R., DAIDONE, M.G., BOLIS, G. & 4 others (1989*b*). Cell kinetics: a prognostic marker in epithelial ovarian cancer. *Gynecol. Oncol.*, **35**, 15.

SIMES, R.J. & ZELEN, M. (1985). Exploratory data analysis and the use of the hazard function for interpreting survival data: an investigator's primer. *J. Clin. Oncol.*, **3**, 1418.

TUBIANA, M., PEJOVIC, M.H., CHAVAUDRA, N., CONTESSO, G. & MALAISE, E.P. (1984). The long-term prognostic significance of the thymidine labeling index in breast cancer. *Int. J. Cancer*, **33**, 441.


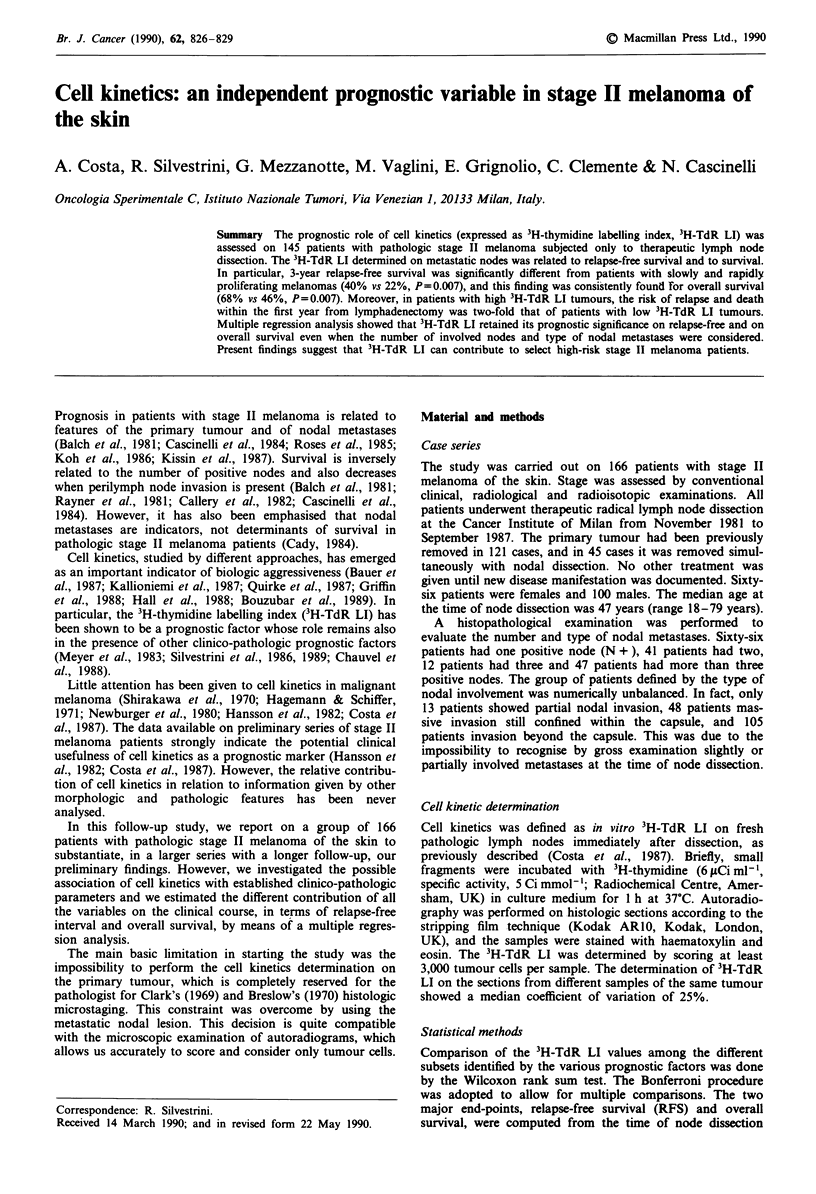

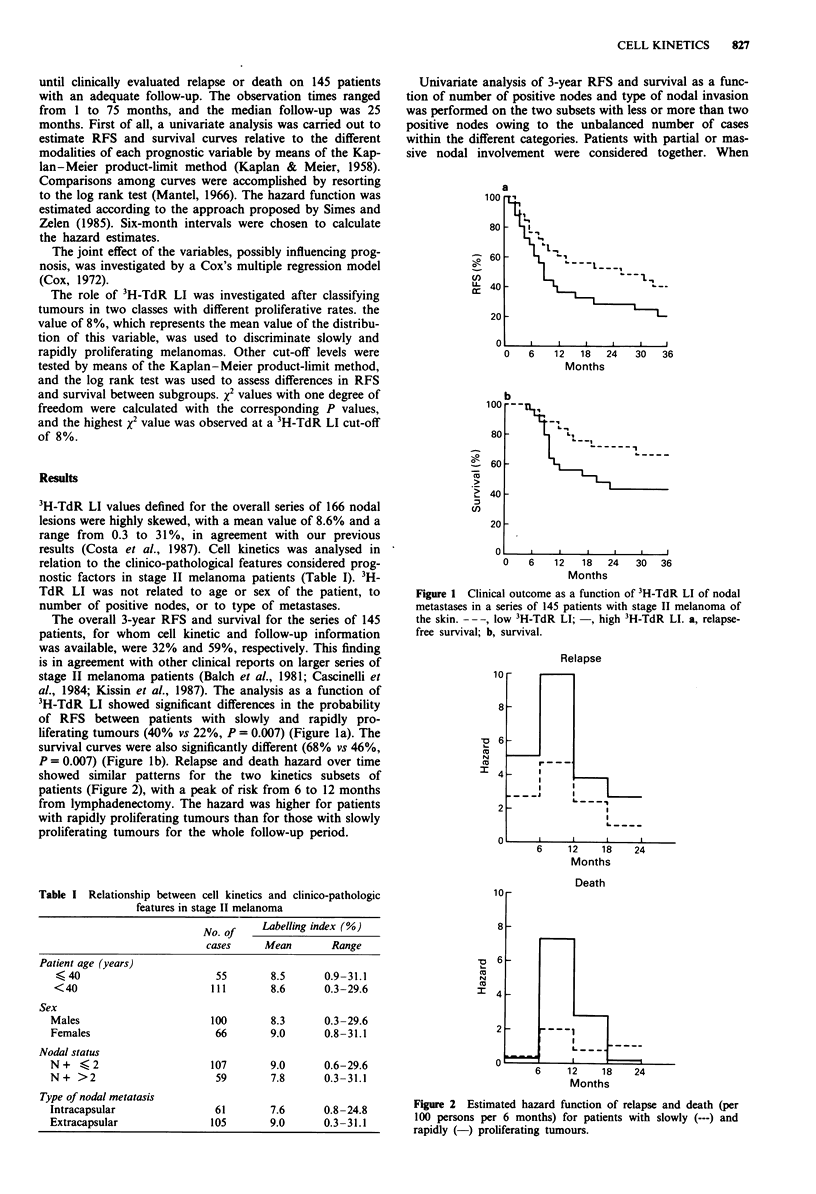

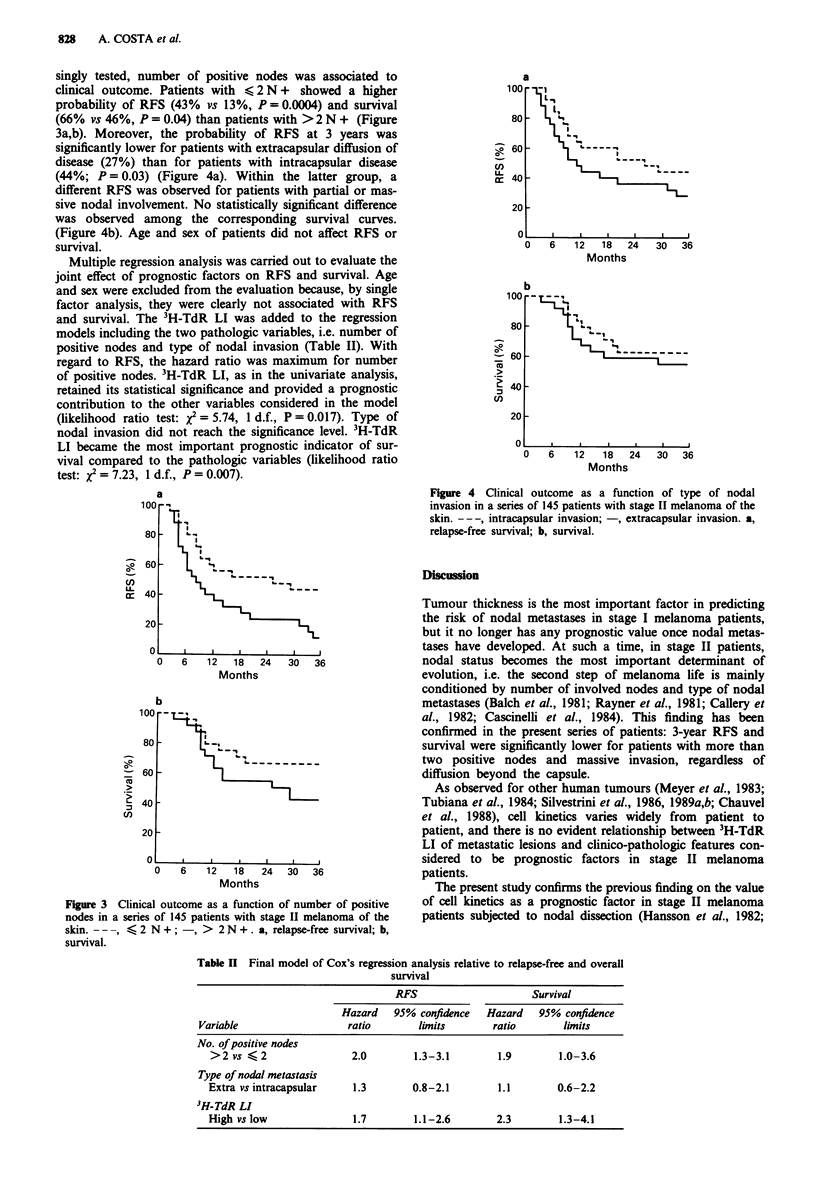

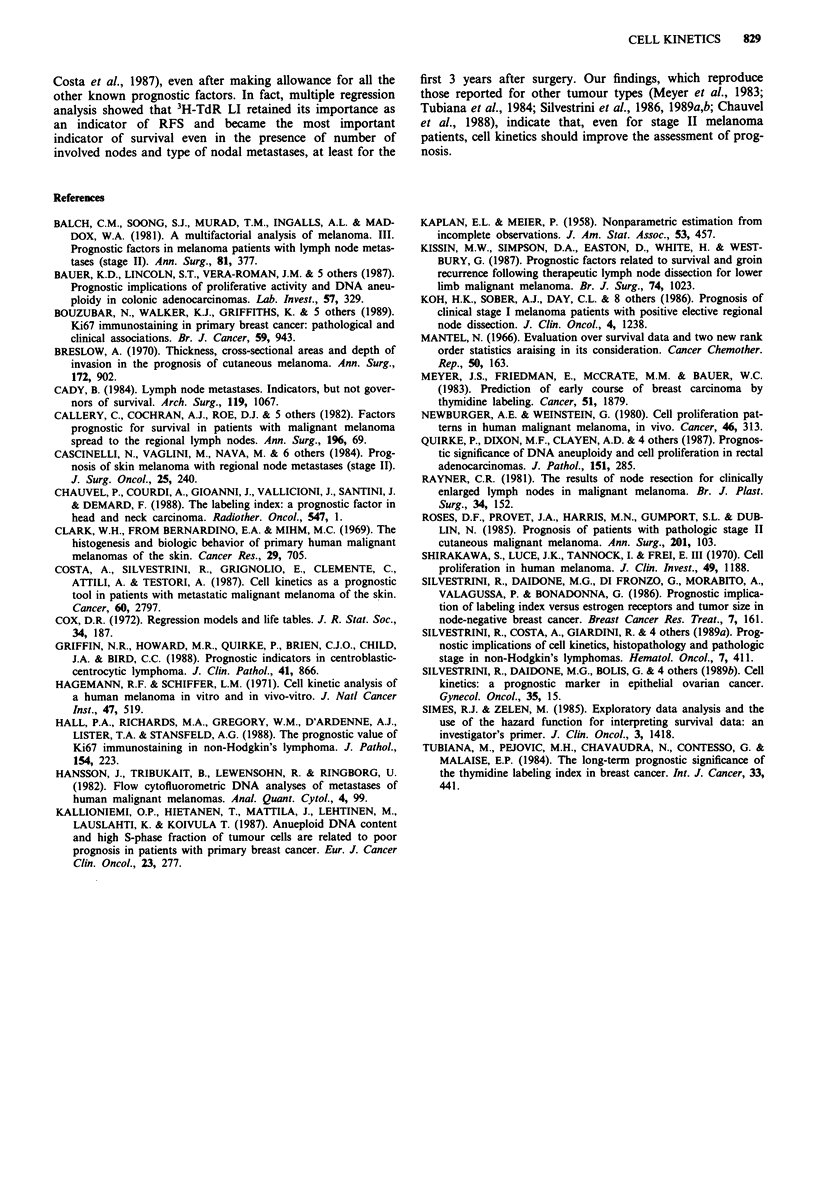


## References

[OCR_00494] Bauer K. D., Lincoln S. T., Vera-Roman J. M., Wallemark C. B., Chmiel J. S., Madurski M. L., Murad T., Scarpelli D. G. (1987). Prognostic implications of proliferative activity and DNA aneuploidy in colonic adenocarcinomas.. Lab Invest.

[OCR_00499] Bouzubar N., Walker K. J., Griffiths K., Ellis I. O., Elston C. W., Robertson J. F., Blamey R. W., Nicholson R. I. (1989). Ki67 immunostaining in primary breast cancer: pathological and clinical associations.. Br J Cancer.

[OCR_00504] Breslow A. (1970). Thickness, cross-sectional areas and depth of invasion in the prognosis of cutaneous melanoma.. Ann Surg.

[OCR_00509] Cady B. (1984). Lymph node metastases. Indicators, but not governors of survival.. Arch Surg.

[OCR_00518] Cascinelli N., Vaglini M., Nava M., Santinami M., Marolda R., Rovini D., Clemente C., Bufalino R., Morabito A. (1984). Prognosis of skin melanoma with regional node metastases (stage II).. J Surg Oncol.

[OCR_00528] Clark W. H., From L., Bernardino E. A., Mihm M. C. (1969). The histogenesis and biologic behavior of primary human malignant melanomas of the skin.. Cancer Res.

[OCR_00533] Costa A., Silvestrini R., Grignolio E., Clemente C., Attili A., Testori A. (1987). Cell kinetics as a prognostic tool in patients with metastatic malignant melanoma of the skin.. Cancer.

[OCR_00543] Griffin N. R., Howard M. R., Quirke P., O'Brien C. J., Child J. A., Bird C. C. (1988). Prognostic indicators in centroblastic-centrocytic lymphoma.. J Clin Pathol.

[OCR_00548] Hagemann R. F., Schiffer L. M. (1971). Cell kinetic analysis of a human melanoma in vitro and in vivo-vitro.. J Natl Cancer Inst.

[OCR_00553] Hall P. A., Richards M. A., Gregory W. M., d'Ardenne A. J., Lister T. A., Stansfeld A. G. (1988). The prognostic value of Ki67 immunostaining in non-Hodgkin's lymphoma.. J Pathol.

[OCR_00559] Hansson J., Tribukait B., Lewensohn R., Ringborg U. (1982). Flow cytofluorometric DNA analyses of metastases of human malignant melanomas.. Anal Quant Cytol.

[OCR_00564] Kallioniemi O. P., Hietanen T., Mattila J., Lehtinen M., Lauslahti K., Koivula T. (1987). Aneuploid DNA content and high S-phase fraction of tumour cells are related to poor prognosis in patients with primary breast cancer.. Eur J Cancer Clin Oncol.

[OCR_00577] Kissin M. W., Simpson D. A., Easton D., White H., Westbury G. (1987). Prognostic factors related to survival and groin recurrence following therapeutic lymph node dissection for lower limb malignant melanoma.. Br J Surg.

[OCR_00581] Koh H. K., Sober A. J., Day C. L., Lew R. A., Kopf A. W., Lamar W., Ben Cosimi A., Wood W. C., Mihm M. C., Malt R. A. (1986). Prognosis of clinical stage I melanoma patients with positive elective regional node dissection.. J Clin Oncol.

[OCR_00586] Mantel N. (1966). Evaluation of survival data and two new rank order statistics arising in its consideration.. Cancer Chemother Rep.

[OCR_00591] Meyer J. S., Friedman E., McCrate M. M., Bauer W. C. (1983). Prediction of early course of breast carcinoma by thymidine labeling.. Cancer.

[OCR_00596] Newburger A. E., Weinstein G. (1980). Cell proliferation patterns in human malignant melanoma, in vivo.. Cancer.

[OCR_00599] Quirke P., Dixon M. F., Clayden A. D., Durdey P., Dyson J. E., Williams N. S., Bird C. C. (1987). Prognostic significance of DNA aneuploidy and cell proliferation in rectal adenocarcinomas.. J Pathol.

[OCR_00604] Rayner C. R. (1981). The results of node resection for clinically enlarged lymph nodes in malignant melanoma.. Br J Plast Surg.

[OCR_00611] Roses D. F., Provet J. A., Harris M. N., Gumport S. L., Dubin N. (1985). Prognosis of patients with pathologic stage II cutaneous malignant melanoma.. Ann Surg.

[OCR_00614] Shirakawa S., Luce J. K., Tannock I., Frei E. (1970). Cell proliferation in human melanoma.. J Clin Invest.

[OCR_00623] Silvestrini R., Costa A., Giardini R., Boracchi P., Del Bino G., Marubini E., Rilke F. (1989). Prognostic implications of cell kinetics, histopathology and pathologic stage in non-Hodgkin's lymphomas.. Hematol Oncol.

[OCR_00618] Silvestrini R., Daidone M. G., Di Fronzo G., Morabito A., Valagussa P., Bonadonna G. (1986). Prognostic implication of labeling index versus estrogen receptors and tumor size in node-negative breast cancer.. Breast Cancer Res Treat.

[OCR_00633] Simes R. J., Zelen M. (1985). Exploratory data analysis and the use of the hazard function for interpreting survival data: an investigator's primer.. J Clin Oncol.

[OCR_00638] Tubiana M., Pejovic M. H., Chavaudra N., Contesso G., Malaise E. P. (1984). The long-term prognostic significance of the thymidine labelling index in breast cancer.. Int J Cancer.

